# Supplementary material for: Evaluation of Bispecific T-Cell Engagers Targeting Murine Cytomegalovirus
Source: Viruses. 2024 May 29;16(6):869. doi: 10.3390/v16060869 (PMC11209133; doi:10.3390/v16060869)
Supplement: Supplementary file 1 [file viruses-16-00869-s001.zip › viruses-3018859-supplementary.pdf]

viruses

Supplementary

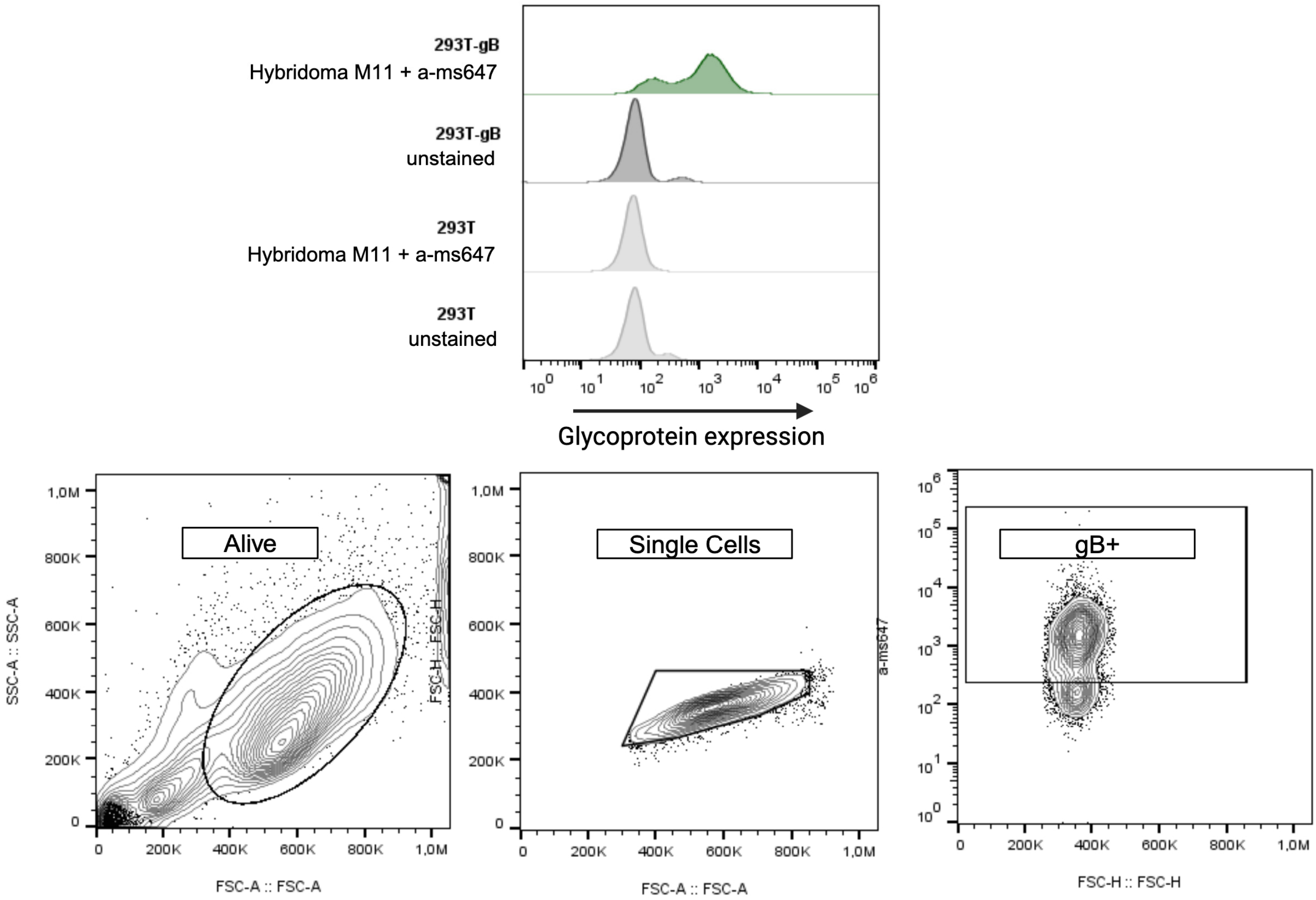

Figure S1. Detailed gating strategy in flow cytometry showed in Figure 4. HEK293T cells expressing gB under anbiotic selection were incubated with hybridoma supernatant and detected using α-mouse Alexa647 antibody. The were measured via flow cytometry. Histogram shows overall fluorescence signal over all controls. Following gating shows selection of glycoprotein-positive, live single target cells. The same strategy was used on HEK293T-gH-gL cells.

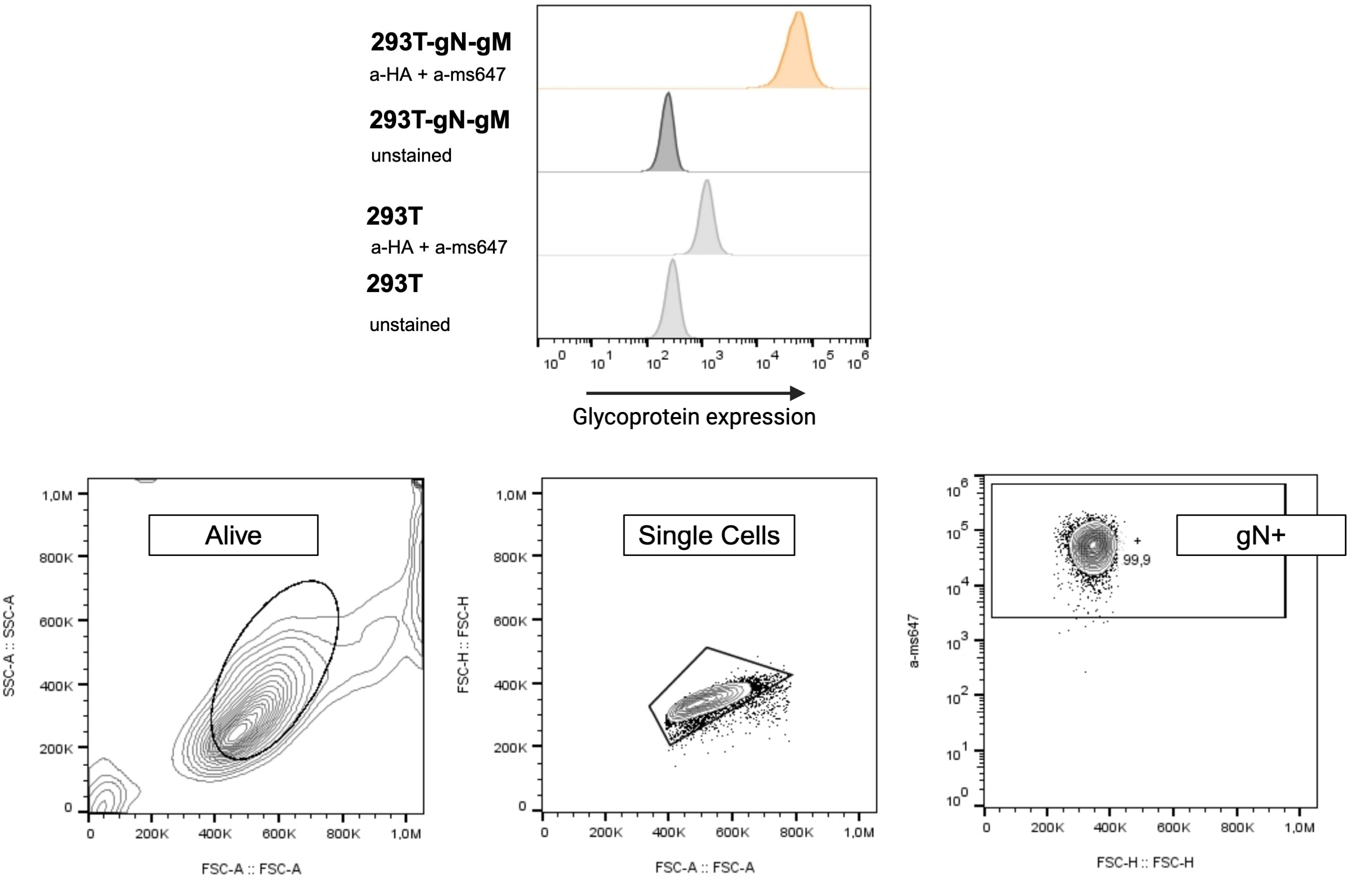

Figure S2. Detailed gating strategy in flow cytometry showed in Figure 4. HEK293T cells expressing gN and gM under anbiotic selection were intracellularly stained with α-HA antibody and detected using α-mouse Alexa647 antibody. The were measured via flow cytometry. Histogram shows overall fluorescence signal over all controls Following gating shows selection of glycoprotein-positive, live single target cells.

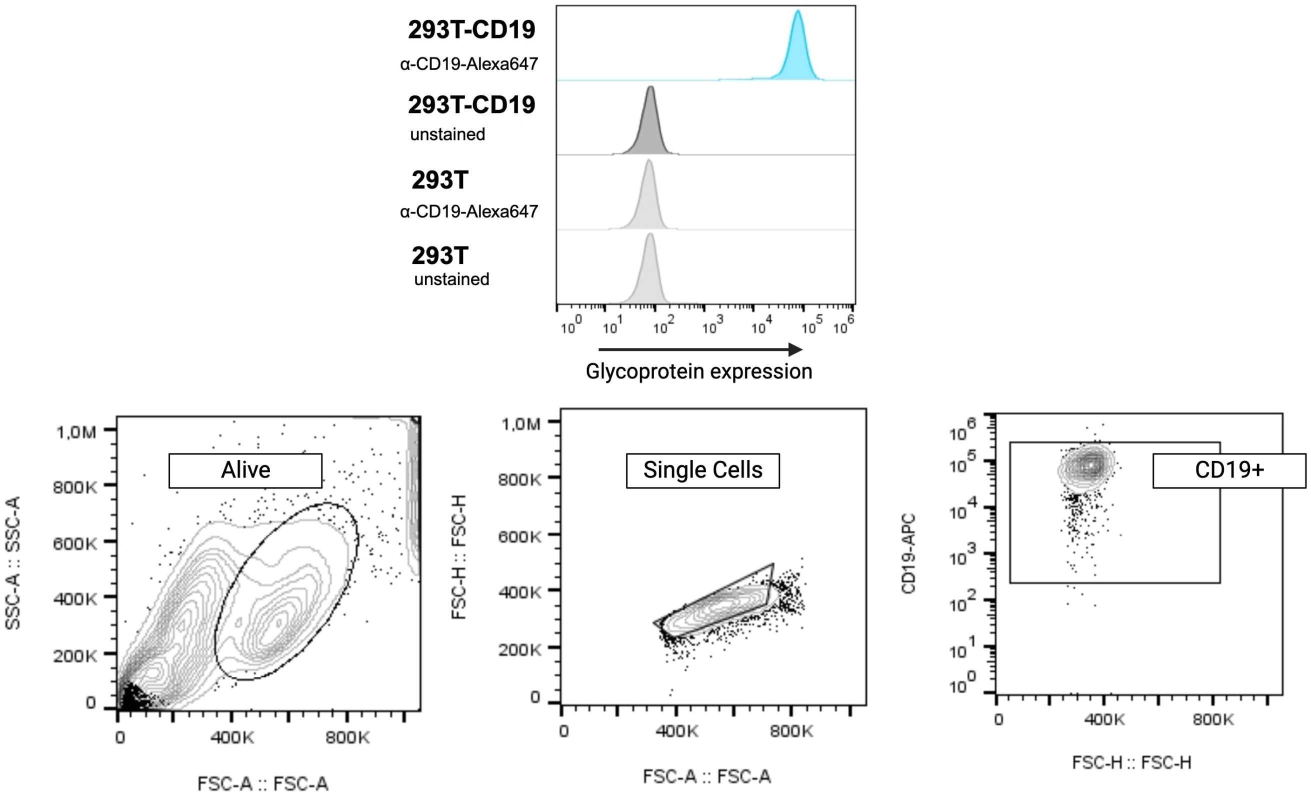

Figure S3. Detailed gating strategy in flow cytometry showed in Figure 4. HEK293T cells expressing CD19 under anbiotic selection were stained and detected with α-CD19-Alexa647 antibody. The were measured via flow cytometry. Histogram shows overall fluorescence signal over all controls. Following gating shows selection of glycoprotein-positive, live single target cells.

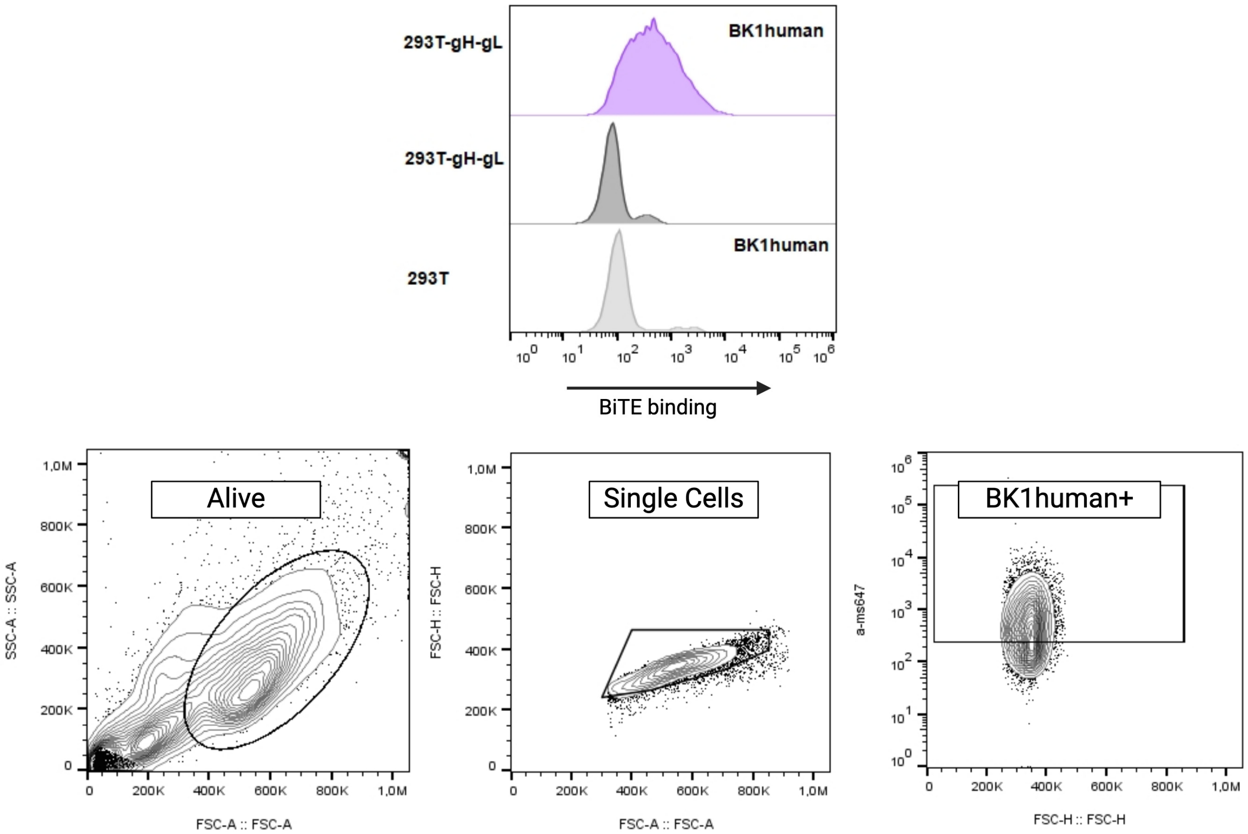

Figure S4. Detailed gating strategy in flow cytometry showed in Figure 5. HEK293T cells expressing gH and gL under anbiotic selection were incubated with BK1human which were then detected using α-Flag and α-mouse Alexa647 antibody. The were measured via flow cytometry. Histogram shows overall fluorescence signal over all controls. Following gating shows selection of live, single target cell bound by BiTE. The same strategy was used to evaluate all BiTE.

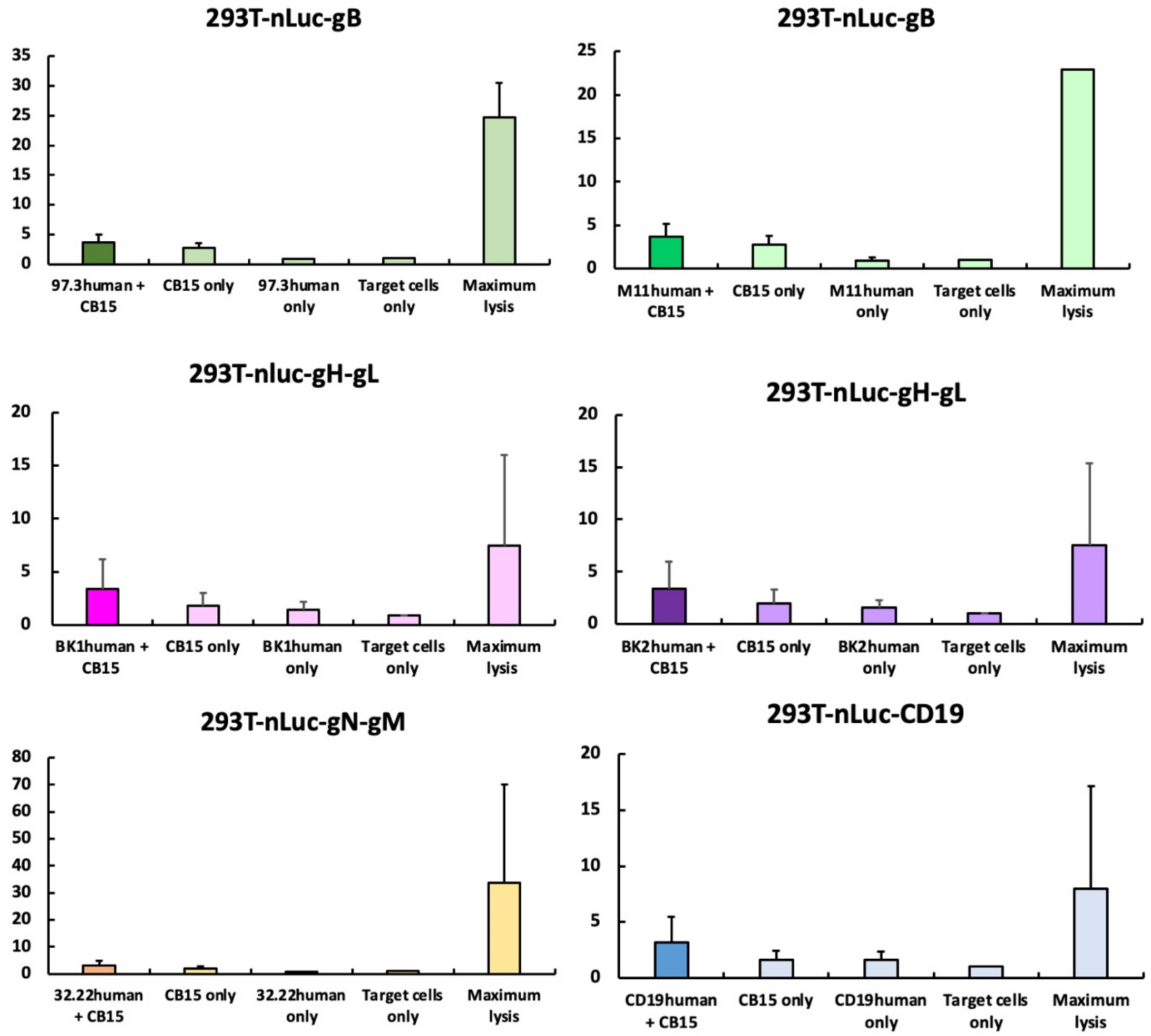

Figure S5. Figure 7 with addition of maximum lysis induced with 0.5% Saponin.

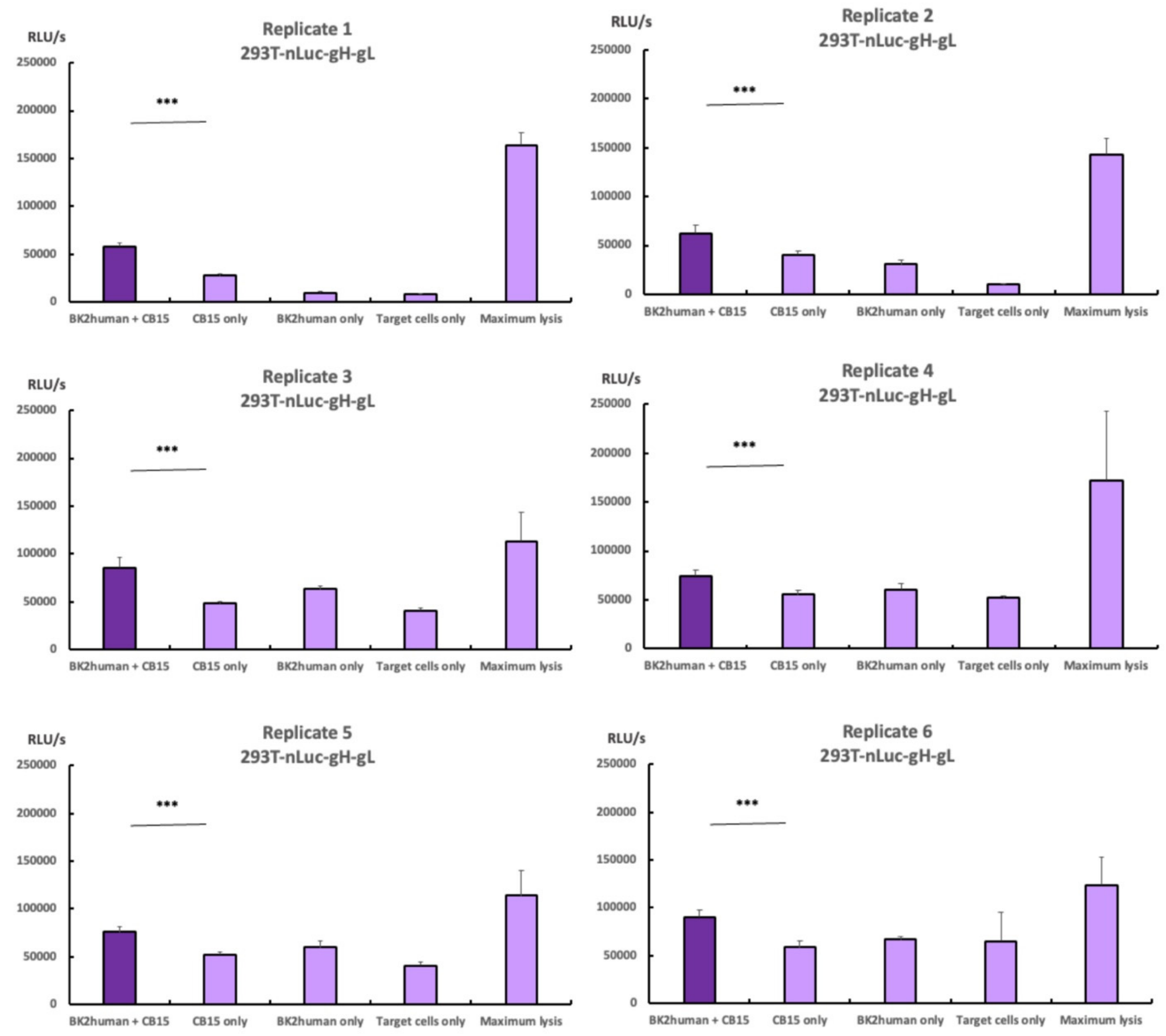

Figure S6. Exemplary depiction of individual biological replicates of nLuc-release assay (BK2human) as seen as normalized dots in Figure 7. HEK293T-nLuc-gH-gL were incubated with CB15 T cells and BiTE. About 24 h later, supernatant was harvested and measured. Maximum lysis was induced using 0.5% Saponin. Columns indicate raw data in RLU/s measured for each individual BiTE containing supernatant (n=6) with 8 technical replicates per control. Error bars indicate standard deviation. P-value was calculated using double sided, unpaired Student's t-test (* = $p < 0.05$; ** = $p < 0.01$; *** = $p < 0.001$).
